# Klotho deficiency and cognitive impairment in dialysis patients: mechanisms, clinical evidence, and therapeutic implications

**DOI:** 10.3389/fnagi.2026.1838285

**Published:** 2026-06-23

**Authors:** Weiwei Hu, Yuke Kong, Jianqin Wang

**Affiliations:** Department of Nephrology, The Second Hospital of Lanzhou University, Lanzhou, China

**Keywords:** chronic kidney disease, cognitive dysfunction, dialysis, inflammatory response, Klotho protein, oxidative stress, pathogenic mechanisms

## Abstract

Cognitive impairment is highly prevalent among patients with end-stage renal disease (ESRD), particularly those undergoing dialysis, and is associated with poor clinical outcomes and reduced quality of life. Emerging evidence suggests that Klotho, an anti-aging protein predominantly expressed in the kidney, plays a critical role in the pathophysiology of uremia-related cognitive dysfunction. This review summarizes current clinical and experimental evidence regarding Klotho deficiency in dialysis patients and its association with cognitive impairment. We further discuss the underlying mechanisms, including oxidative stress, chronic inflammation, vascular dysfunction, and blood–brain barrier disruption. In addition, the potential role of Klotho as a biomarker and therapeutic target is critically evaluated. Understanding the kidney–brain axis mediated by Klotho may provide novel insights into early identification and targeted intervention for cognitive impairment in dialysis populations.

## 1 Introduction

Chronic kidney disease (CKD), particularly at advanced stages leading to uremia, is a major global health burden characterized by progressive renal dysfunction and multisystem complications. Cognitive impairment is increasingly recognized as a common and clinically significant complication in patients with CKD and end-stage renal disease (ESRD), especially those undergoing dialysis. Epidemiological studies estimate that 20%−70% of these patients exhibit cognitive decline, ranging from mild cognitive impairment to dementia, and this is associated with poor treatment adherence, increased hospitalization, and higher mortality (Andrews et al., 2025). The mechanisms underlying cognitive impairment in CKD are complex and multifactorial, involving uremic toxin accumulation, chronic inflammation, oxidative stress, endothelial dysfunction, and disturbances in mineral metabolism (Ghoshal, 2023). The concept of the “kidney–brain axis” highlights the bidirectional interactions between renal dysfunction and cerebral injury, including blood–brain barrier disruption, neuroinflammation, and microvascular damage (Ghoshal, 2023; Bobot et al., 2020). Despite advances in dialysis therapy, cognitive impairment remains under-recognized and inadequately managed, underscoring the need to identify key molecular mediators. Klotho, an anti-aging protein predominantly expressed in the kidney, declines early in CKD and is implicated in vascular, metabolic, and neuroprotective regulation (Prud'homme et al., 2022; Rodríguez-Ortiz et al., 2024). Notably, Klotho is a key regulator of phosphate homeostasis through the FGF23–Klotho axis. Early Klotho deficiency impairs phosphaturia and promotes compensatory FGF23 elevation; however, progressive CKD leads to phosphate retention even before overt hyperphosphatemia develops (Prud'homme et al., 2022; Huang et al., 2022; Yamada and Giachelli, 2017). Emerging evidence suggests that Klotho deficiency may represent a key mechanistic link between renal dysfunction and cognitive impairment. Uremic toxins, hyperphosphatemia, and vascular calcification contribute to cerebral injury, while Klotho deficiency exacerbates these processes (Bobot et al., 2020; Bobot, 2023; Li et al., 2025). Therefore, this review summarizes current evidence on the role of Klotho in dialysis-related cognitive impairment, focusing on underlying mechanisms and its potential as a biomarker and therapeutic target. In addition, this review integrates clinical, molecular, and genetic evidence to provide a comprehensive framework linking Klotho deficiency to cognitive dysfunction in dialysis patients.

## 2 Biological characteristics of Klotho protein and its renal expression

### 2.1 Structure and isoforms of Klotho

Klotho exists in two main forms: membrane-bound Klotho (mKlotho) and soluble Klotho (sKlotho), which exert complementary biological functions. mKlotho is predominantly expressed in the kidney and functions as a co-receptor for fibroblast growth factor 23 (FGF23), thereby regulating phosphate metabolism. Disruption of the FGF23–Klotho axis contributes to hyperphosphatemia and vascular calcification, which are common in CKD (Zhong et al., 2020; Liu et al., 2022), while sKlotho is generated through proteolytic shedding or alternative splicing and is detectable in circulation. It acts as an endocrine factor with antioxidative and anti-inflammatory properties and modulates multiple signaling pathways beyond the kidney (Chen et al., 2020). The balance between mKlotho and sKlotho is essential for maintaining mineral metabolism and vascular homeostasis, and its disruption contributes to systemic complications in CKD.

### 2.2 Renal expression and regulation of Klotho

Klotho is predominantly expressed in the distal tubules of the kidney, which serve as the primary source of circulating sKlotho. In CKD, Klotho expression declines early and progressively with disease severity, and this reduction contributes to renal dysfunction rather than being merely a consequence (Li et al., 2023; Kale et al., 2023). Klotho expression is regulated by multiple factors, including inflammation, oxidative stress, and epigenetic modifications. Pro-inflammatory cytokines suppress Klotho transcription via NF-κB signaling, while oxidative stress reduces Klotho expression at transcriptional and post-translational levels (Li et al., 2023; Valiño-Rivas et al., 2022). Epigenetic mechanisms, such as histone deacetylation, further contribute to gene silencing but may be reversible (Xia and Cao, 2021; Kale et al., 2022). In addition, genetic polymorphisms and transcription factors such as Sp1 influence Klotho expression, contributing to inter-individual variability in CKD progression (Xu et al., 2021; Li et al., 2020).

### 2.3 Systemic functions of Klotho

Klotho exerts broad protective effects across multiple organ systems. It reduces oxidative stress through activation of the Nrf2 pathway and suppresses inflammation by inhibiting pro-inflammatory signaling cascades (Zhao et al., 2022; Nambidi et al., 2025). Klotho also modulates Wnt/β-catenin signaling, thereby attenuating fibrosis and maintaining tissue integrity (Kadoya et al., 2020). In the nervous system, Klotho supports neuronal survival, enhances synaptic function, and reduces neuroinflammation, contributing to preservation of cognitive function (Hanson et al., 2021). In addition, Klotho regulates metabolic homeostasis by modulating insulin and fibroblast growth factor signaling, influencing glucose metabolism and energy balance (Landry et al., 2021).

### 2.4 Klotho and chronic kidney disease

Klotho deficiency is a feature of CKD and contributes to mineral metabolism disorders, vascular calcification, and renal fibrosis (Wang et al., 2024). Emerging evidence suggests that circulating Klotho levels may vary according to the underlying etiology of CKD. In particular, patients with diabetic kidney disease (DKD) exhibit more pronounced reductions in Klotho expression compared with non-diabetic CKD, likely due to the combined effects of hyperglycemia, oxidative stress, advanced glycation end products, and chronic inflammation (Yang et al., 2020). These metabolic disturbances suppress renal Klotho expression and accelerate endothelial dysfunction, vascular injury, and renal fibrosis. In contrast, other CKD etiologies, such as glomerulonephritis and hypertensive nephropathy, may demonstrate different patterns and rates of Klotho decline depending on disease mechanisms and progression (Guo et al., 2024). This variability suggests that circulating Klotho levels reflect not only renal function decline but also disease-specific pathophysiological processes. Importantly, because diabetes is an established risk factor for both vascular cognitive impairment and neurodegeneration, the more severe Klotho deficiency observed in DKD may partially contribute to the increased cognitive burden in this population (Edmonston et al., 2024; The et al., 2024). Furthermore, metabolic and vascular abnormalities associated with diabetes may amplify the detrimental effects of Klotho deficiency on the kidney–brain axis. Overall, these findings indicate that the nature of kidney disease may influence circulating Klotho levels and potentially contribute to heterogeneity in cardiovascular and neurocognitive outcomes among CKD patients.

## 3 Clinical association between Klotho protein and cognitive dysfunction in uremic patients

### 3.1 Epidemiology of cognitive impairment in uremia

Cognitive impairment is highly prevalent in patients with uremia, particularly those undergoing dialysis. Approximately 60%−75% of hemodialysis patients exhibit cognitive dysfunction, ranging from mild impairment to dementia, and this is associated with reduced quality of life, poor treatment adherence, and increased mortality (Yu et al., 2021). The etiology is multifactorial, involving uremic toxin accumulation, chronic inflammation, oxidative stress, vascular disease, and metabolic disturbances. Dialysis-related factors, including hemodynamic instability and cerebral hypoperfusion, further contribute to neurovascular injury (Kelly et al., 2021). Clinically, cognitive impairment affects multiple domains, particularly memory, executive function, and attention. Depression frequently coexists and worsens cognitive outcomes. Neuroimaging studies demonstrate hippocampal atrophy and microvascular injury, supporting a central role of neurovascular dysfunction in uremia-associated cognitive decline (Sacchi et al., 2025).

### 3.2 Alterations in Klotho levels in uremic patients

Klotho levels are significantly reduced in patients with CKD and uremia, both in serum and cerebrospinal fluid, reflecting impaired renal production and systemic inflammation (Sacchi et al., 2025). Lower Klotho concentrations are consistently associated with poorer cognitive performance, suggesting a potential role as a biomarker of cognitive impairment (Shibata et al., 2025). These findings support the hypothesis that Klotho deficiency contributes to neurocognitive dysfunction in uremia.

### 3.3 Klotho as a biomarker of cognitive impairment

Klotho has emerged as a potential biomarker for cognitive impairment in CKD and related conditions. Higher circulating Klotho levels are generally associated with better cognitive performance, whereas reduced levels are linked to mild cognitive impairment and adverse neurological outcomes (Zhang and Zhang, 2023; Zhang et al., 2023). Standardized cognitive assessment tools, including the Montreal Cognitive Assessment (MoCA) and the Mini-Mental State Examination (MMSE), are widely used in CKD populations and are sensitive for detecting early cognitive decline (Aggarwal et al., 2020). Several studies have consistently demonstrated lower MoCA and MMSE scores in patients with CKD and those undergoing hemodialysis compared with healthy controls, highlighting the substantial burden of cognitive dysfunction in this population.

Emerging evidence further supports an association between circulating α-Klotho levels and cognitive performance. Observational studies have shown that lower serum Klotho concentrations are independently associated with worse cognitive outcomes after adjustment for confounding factors, suggesting a potential role of Klotho deficiency in cognitive dysfunction (Zhang and Zhang, 2023). Population-based analyses additionally indicate that higher Klotho levels correlate with better performance across multiple cognitive domains, including memory and executive function (Sacchi et al., 2025). However, studies directly integrating serum Klotho measurements with standardized cognitive assessments such as MoCA or MMSE within the same CKD cohort remain limited. Therefore, although existing evidence supports a potential clinical relationship between Klotho deficiency and cognitive impairment, further prospective studies are needed to clarify whether circulating sKlotho independently predicts cognitive decline in CKD and dialysis populations. Accurate measurement of circulating Klotho also remains a major methodological challenge. Enzyme-linked immunosorbent assay (ELISA) is currently the most widely used method in clinical studies because of its convenience and scalability. Nevertheless, accumulating evidence suggests that ELISA-based assays may exhibit limited specificity and substantial inter-assay variability, potentially contributing to inconsistent findings across studies. In contrast, immunoprecipitation–immunoblotting (IP–IB) is considered the gold standard method for soluble Klotho measurement because of its superior specificity and more reliable detection of full-length Klotho protein (Neyra et al., 2020). Variability among assay platforms may therefore partly explain the heterogeneity observed in studies evaluating the association between circulating Klotho levels and cognitive outcomes (Sacchi et al., 2025; Zhang and Zhang, 2023).

Taken together, current evidence supports the potential utility of Klotho as a biomarker for CKD-related cognitive impairment. However, large-scale prospective studies incorporating standardized cognitive assessments, validated Klotho measurement techniques, and robust multivariable analyses are needed to determine whether Klotho independently predicts cognitive decline in CKD and dialysis populations.

### 3.4 Klotho, serum phosphate, and cerebrovascular pathology in dialysis patients

Cerebrovascular disease is a major contributor to cognitive impairment in dialysis patients. Klotho plays a significant role in vascular homeostasis through regulation of mineral metabolism, oxidative stress, endothelial function, and phosphate balance. Reduced Klotho levels are associated with vascular calcification, a key contributor to cerebrovascular injury in CKD (Konnur et al., 2024). Lower serum Klotho concentrations have also been independently associated with carotid artery calcification in dialysis patients (Guo et al., 2021). In addition, Klotho exerts antioxidant and vasculoprotective effects that help preserve endothelial and microvascular injury. Serum phosphate may represent a key mediator linking Klotho deficiency to dementia risk in CKD and dialysis populations (Lisowska et al., 2022; Adamska-Tomaszewska et al., 2020). Phosphate retention occurs early in CKD progression and is tightly regulated by the FGF23–Klotho axis. Declining renal Klotho expression reduces phosphaturia and triggers compensatory FGF23 elevation to maintain phosphate homeostasis (Han, 2015). However, progressive nephron loss and persistent Klotho deficiency ultimately impair phosphate excretion, leading to chronic phosphate overload and CKD–mineral bone disorder (CKD-MBD). Elevated serum phosphate contributes not only to vascular calcification but also to neurovascular and neurodegenerative injury (Ishida et al., 2021). Hyperphosphatemia promotes endothelial dysfunction, oxidative stress, inflammation, and osteogenic transformation of vascular smooth muscle cells, thereby accelerating arterial stiffness and vascular calcification. These changes impair cerebral perfusion and contribute to cerebral small vessel disease, white matter lesions, lacunar infarction, and chronic cerebral hypoperfusion, which are major substrates of vascular cognitive impairment in dialysis patients (Li et al., 2017). Elevated phosphate levels have also been associated with increased carotid intima–media thickness and intracranial vascular calcification, both linked to dementia risk. Beyond vascular injury, phosphate toxicity may exert direct neurotoxic effects. Experimental studies suggest that excessive phosphate disrupts blood–brain barrier integrity, activates neuroinflammatory pathways, and exacerbates neuronal injury. Given the antioxidative and anti-inflammatory properties of Klotho, Klotho deficiency may further amplify phosphate-mediated cerebrovascular damage and synaptic dysfunction. Elevated circulating FGF23 levels have also been associated with impaired cognition and increased cerebrovascular risk, although whether these effects are mediated directly or indirectly through phosphate dysregulation remains unclear.

Clinical evidence further supports this association. Population-based studies have shown that higher serum phosphorus levels are independently associated with increased dementia risk. In CKD and dialysis cohorts, elevated serum phosphate correlates with worse cognitive performance, suggesting that chronic phosphate retention contributes to progressive neurocognitive decline (Vazquez-Sanchez et al., 2023). Dialysis initiation and kidney transplantation may also influence Klotho-related neurovascular pathology. Although dialysis partially corrects the uremic milieu, circulating Klotho levels generally remain low, and neurovascular risk often persists despite renal replacement therapy. Hemodynamic instability, recurrent cerebral hypoperfusion, oxidative stress, and chronic inflammation during hemodialysis may further contribute to endothelial dysfunction and cognitive decline. Dialysis modality, treatment adequacy, and residual renal function may also affect circulating Klotho levels and vascular outcomes. In contrast, kidney transplantation has been associated with partial restoration of circulating Klotho levels and improvement in mineral metabolism, vascular function, and inflammatory status. Transplantation may also attenuate vascular calcification and endothelial dysfunction, potentially contributing to better neurocognitive outcomes than long-term dialysis. Restoration of renal Klotho expression after transplantation may partially correct abnormalities in the Klotho–FGF23–phosphate axis and reduce neurovascular injury. However, current evidence remains largely observational, and whether improvements in cognition and cerebrovascular function are directly mediated by Klotho restoration remains unclear.

Overall, current evidence suggests that serum phosphate is not merely a biochemical marker of CKD-MBD, but a potential pathogenic mediator linking Klotho deficiency, FGF23 dysregulation, cerebrovascular injury, and dementia development (Viggiano et al., 2020). Therefore, early phosphate control and restoration of Klotho homeostasis may help preserve cognitive function in CKD and dialysis populations, although further longitudinal and interventional studies are needed.

## 4 Molecular mechanisms linking Klotho deficiency to cognitive impairment

### 4.1 Oxidative stress and neuroprotection

Oxidative stress is a central mechanism underlying cognitive impairment in uremia. Excessive accumulation of reactive oxygen species (ROS) leads to lipid peroxidation, DNA damage, synaptic dysfunction, and neuronal apoptosis. Klotho plays a key role in maintaining redox homeostasis and protecting neuronal integrity. Experimental studies demonstrate that Klotho enhances antioxidant defenses through activation of the Nrf2 pathway and upregulation of antioxidant enzymes such as MnSOD, thereby reducing oxidative damage in the hippocampus and improving cognitive function (Karizmeh et al., 2022). Conversely, Klotho deficiency is associated with increased oxidative stress and neuronal apoptosis, contributing to cognitive decline (Nambidi et al., 2025; Youssef et al., 2020). Klotho also regulates nitric oxide synthase activity, reducing nitrosative stress and preserving vascular and neuronal function (Olejnik et al., 2025). Given that oxidative stress is amplified in CKD due to uremic toxins and chronic inflammation, reduced Klotho levels may exacerbate neuronal injury and accelerate cognitive impairment in dialysis patients.

### 4.2 Inflammation and immune dysregulation

Chronic low-grade inflammation is a mark of CKD and a key driver of cognitive dysfunction. Klotho exerts anti-inflammatory effects primarily through inhibition of the NF-κB signaling pathway, thereby reducing the production of pro-inflammatory cytokines such as TNF-α, IL-1β, and IL-6 (Wei et al., 2025). Klotho deficiency enhances NF-κB activation and promotes systemic and neuroinflammation, leading to blood–brain barrier disruption, synaptic dysfunction, and neuronal apoptosis. In addition, Klotho suppresses activation of the NLRP3 inflammasome, further limiting inflammatory injury (Olejnik et al., 2025). Clinical evidence shows an inverse relationship between circulating Klotho levels and inflammatory markers, indicating that Klotho deficiency contributes to a pro-inflammatory state in CKD (Liang et al., 2025; Jia et al., 2024). This persistent inflammation plays a critical role in cognitive decline in uremic patients.

### 4.3 Vascular dysfunction and cerebral hypoperfusion

Vascular dysfunction is a major contributor to cognitive impairment in CKD (Elmahalawy and Farres, 2024). Klotho plays a critical role in maintaining vascular homeostasis by preserving endothelial function, reducing oxidative stress, inhibiting vascular calcification, and regulating phosphate metabolism. Reduced Klotho levels are associated with endothelial dysfunction, arterial stiffness, and phosphate retention, all of which impair cerebral blood flow and promote microvascular injury (Bi et al., 2020; Zavvari et al., 2025). Hyperphosphatemia, which develops early as a consequence of Klotho–FGF23 axis dysregulation, further accelerates vascular calcification and endothelial injury, thereby contributing to cerebral small vessel disease and chronic cerebral hypoperfusion (Zavvari et al., 2025; Hellou et al., 2025).

### 4.4 Neurodegeneration and synaptic dysfunction

Klotho plays a significant role in maintaining neuronal survival and synaptic function. It enhances synaptic plasticity and supports neuronal signaling, partly through modulation of NMDA receptor activity (Hanson et al., 2021; Castner et al., 2023). Klotho also attenuates neurodegeneration by reducing oxidative stress and inflammation. It suppresses activation of the NLRP3 inflammasome and decreases pro-inflammatory cytokine production, thereby protecting neurons from injury (Xiang et al., 2022). Moreover, Klotho influences amyloid metabolism by promoting clearance of β-amyloid and reducing its accumulation, which is relevant to neurodegenerative diseases such as Alzheimer's disease (Fung et al., 2022). Reduced Klotho levels are associated with increased amyloid burden, tau pathology, and cognitive decline (Sacchi et al., 2025; Yalcin et al., 2025). Furthermore, Klotho deficiency disrupts intracellular signaling pathways, including PI3K/AKT and Wnt signaling, leading to impaired neuronal survival and synaptic dysfunction. These alterations contribute to progressive neurodegeneration and cognitive impairment in CKD.

Overall, klotho deficiency contributes to cognitive impairment through interconnected mechanisms involving oxidative stress, chronic inflammation, vascular dysfunction, and neurodegeneration. These pathways form a mechanistic network linking renal dysfunction to brain injury. Targeting Klotho-related pathways may provide a promising strategy to mitigate cognitive decline in patients with CKD and uremia.

## 5 Genetic polymorphisms of Klotho and cognitive dysfunction in uremic patients

### 5.1 Common Klotho polymorphisms and functional effects

Several single nucleotide polymorphisms (SNPs) in the Klotho gene influence its expression and biological activity, thereby potentially affecting susceptibility to CKD and cognitive impairment. Among these, the G395A polymorphism (rs1207568) located in the promoter region regulates transcriptional activity and circulating Klotho levels. The A allele has been associated with altered Klotho expression and increased risk of metabolic and vascular disorders, which are closely linked to CKD progression and cognitive decline (Jiang et al., 2024; Helvaci et al., 2021). The C1818T polymorphism, located in exon 4, appears to exert more modest or context-dependent effects. Although its independent association with disease outcomes is inconsistent, it may contribute to complex phenotypes when combined with other variants, suggesting a role for haplotype interactions. The KL-VS haplotype (rs9536314 and rs9527025) is the most extensively studied variant in aging and neurocognitive research. KL-VS heterozygosity has been associated with improved cognitive performance and reduced neurodegeneration in some populations, possibly through enhanced Klotho activity (Driscoll et al., 2021). However, findings are inconsistent, indicating that its functional effects are influenced by population characteristics and disease context.

### 5.2 Clinical studies on Klotho gene polymorphisms and cognitive dysfunction

Clinical evidence suggests that Klotho polymorphisms may influence cognitive outcomes, although results remain heterogeneous. Some studies report that G395A variants are associated with increased risk of CKD, but their direct relationship with cognitive impairment is less consistent (Gunawan et al., 2020). In non-CKD populations, KL-VS heterozygosity has been linked to domain-specific cognitive effects, suggesting that Klotho variants may differentially affect cognitive functions depending on disease status and population characteristics (Majumdar et al., 2025). Population heterogeneity plays an important role, as differences in allele frequency, environmental exposure, and comorbidities may contribute to variable findings. Overall, current evidence supports a potential association between Klotho genetics and cognition, but causality remains unclear.

### 5.3 Klotho and interactions with other genetic markers

Klotho polymorphisms may interact with other genetic variants to influence CKD progression and cognitive decline. For example, variants in the nitric oxide synthase gene (NOS3), which regulates vascular function, have been associated with both CKD and cognitive impairment. The combined effects of NOS3 and Klotho variants may modulate endothelial function and cerebral perfusion, thereby affecting cognitive outcomes (Gunawan et al., 2020). In addition, polymorphisms in inflammatory genes such as IL6 and MIF may amplify the effects of Klotho deficiency by promoting systemic inflammation, a key driver of cognitive impairment in uremia (Guarneri et al., 2022). Interactions with cognition-related genes, particularly apolipoprotein E (APOE), further highlight the complexity of genetic influences. While APOE ε4 is a strong determinant of neurodegeneration, Klotho variants appear to exert modulatory rather than independent effects on cognitive decline (Shibata et al., 2025).

### 5.4 Gene-environment interaction effects on Klotho function

Environmental factors play a crucial role in regulating Klotho expression and function. Chronic inflammation and oxidative stress, which are common in CKD, downregulate Klotho expression and exacerbate cognitive impairment. Environmental toxins such as cadmium have been shown to reduce Klotho expression through activation of stress-related signaling pathways, thereby contributing to neurotoxicity and cognitive dysfunction (Liu et al., 2023). Epigenetic mechanisms, including DNA methylation of the Klotho promoter, further mediate environmental influences on gene expression (Kawarazaki and Fujita, 2021). Lifestyle and metabolic factors may also modulate Klotho activity. Interventions targeting oxidative stress, inflammation, and epigenetic regulation have the potential to restore Klotho expression and mitigate disease progression.

In conclusion, Klotho gene polymorphisms contribute to inter-individual variability in CKD progression and cognitive outcomes. However, their effects are complex and influenced by gene–gene and gene–environment interactions. Although Klotho genetics shows promise for risk stratification, current evidence is limited and inconsistent. Further large-scale and longitudinal studies are needed to clarify its clinical relevance in uremia-associated cognitive impairment.

## 6 Clinical implications and future directions

Despite growing evidence linking Klotho deficiency to cognitive impairment in dialysis patients, several limitations remain. Most studies are observational or experimental, and causal relationships have not been established. Inconsistent findings regarding circulating Klotho levels may reflect differences in assay methods, patient characteristics, and dialysis modalities. In particular, the lack of standardized methods for soluble Klotho measurement limits its clinical utility as biomarker. Furthermore, it remains unclear whether peripheral Klotho accurately reflect central nervous system activity. From a therapeutic perspective, strategies aimed at restoring Klotho expression—including phosphate control, vitamin D analogs, and recombinant Klotho supplementation—have shown promise in preclinical studies, but robust evidence from large-scale clinical trials is lacking. Future research should move beyond cross-sectional associations toward longitudinal and mechanistic studies to better define causal pathways within the kidney–brain axis.

Importantly, future studies should integrate circulating Klotho measurements with standardized cognitive assessments, such as the MoCA and the MMSE, in longitudinal CKD cohorts. This may improve characterization of the relationship between Klotho deficiency and cognitive decline and help establish Klotho as a clinically relevant biomarker.

In addition, multimodal approaches incorporating neuroimaging, vascular function assessment, and multi-omics analyses may further clarify the interplay among Klotho deficiency, neurovascular dysfunction, and neurodegeneration. Such strategies could help identify high-risk subgroups and support development of targeted interventions. Ultimately, a deeper understanding of the Klotho-mediated kidney–brain axis may facilitate precision medicine approaches and improve cognitive in CKD and dialysis populations.

## 7 Conclusion

Klotho deficiency represents a critical molecular link between chronic kidney disease and cognitive impairment in uremic patients. Accumulating evidence indicates that reduced Klotho levels contribute to cognitive decline through interconnected mechanisms, including oxidative stress, inflammation, vascular dysfunction, phosphate dysregulation, and neurodegeneration. Emerging data further suggest that serum phosphate may function as an important mediator linking Klotho deficiency, FGF23 dysregulation, cerebrovascular injury, and dementia development. Because phosphate retention occurs early in CKD and contributes to vascular calcification, endothelial dysfunction, neuroinflammation, and cerebral hypoperfusion, phosphate overload may represent a modifiable risk factor for cognitive decline in dialysis populations. Targeting the Klotho–FGF23–phosphate axis may therefore provide a novel therapeutic strategy. However, current evidence remains largely observational, and further longitudinal and interventional studies are required to clarify causality and determine optimal treatment strategies.
